# Determination of chlorantraniliprole 18.5% SC in the paddy ecosystem and its risk assessment

**DOI:** 10.1038/s41598-023-32422-w

**Published:** 2023-04-04

**Authors:** Saraswati Mahato, R. Harischandra Naik, M. Bheemanna, M. S. Pallavi, Sujay Hurali, Saroja Narsing Rao, M. Nagaraj Naik, M. Paramsivam

**Affiliations:** 1 Pesticide Residue and Food Quality Analysis Laboratory, University of Agricultural Sciences, Raichur, Karnataka 584 104 India; 2 College of Horticulture, Bangalore, University of Horticultural Sciences, Bagalkot, India; 3 Pesticide Toxicology Laboratory, Tamil Nadu Agricultural University, Coimbatore, Tamil Nadu 641003 India

**Keywords:** Environmental chemistry, Environmental impact, Environmental sciences, Chemistry

## Abstract

Chlorantraniliprole belongsto theanthranilic diamide group is widely used against broad range of lepidopteron pests in a variety of vegetable and rice pests includingyellow rice stem borer and leaf folder. Supervised field trials were conducted during*Rabi* (2018–2019) and *Kharif* (2019) to evaluate the dissipation pattern and risk assessment of chlorantraniliprole 18.5% SC in paddy ecosystem following foliar application at 30 and 60 g a.i. ha^-1^ in two different cropping seasons.Modified QuEChERS (Quick, Easy, Cheap, Effective, Rugged and Safe) technique was used for the extraction of CAP residues with acetonitrile and determined by LC–MS/MS (ESI +).The limit of quantification (LOQ) was 0.01 µg g^−1^ for paddy leaf, straw, husk, and brown rice, respectively and 0.005 µg g^−1^ for soil. The average recoveries obtained were 84.30–88.92% from paddy leaf, 94.25–97.81% from straw, 90.21–93.38% from husk, 93.57–96.40% from brown rice and 89.93–91.14% from soil. The residues in paddy leaf dissipated within 35–40 days with a half-life of 4.33–5.07 days in *Rabi* and 3.92–4.86 days in *Kharif* at 30 and 60 g a.i. ha^−1^, respectively. The residues in soil dissipated within 15–21 days with a half-life of 14.44–15.75 days in *Rabi* and 13.33–14.44 days in *Kharif* at respective doses. At harvest chlorantraniliprole residues were not detected in straw, husk, and brown rice. The dietary risk of paddy leaf (green fodder) for cattle was found safe for consumption as the hazard index is less than one. Soil ecological risk assessment was found to be less than one (RQ < 0.1) for earthworms (*Eisenia foetida*) and arthropods (*Aphidiusrhopalosiphi*). The presentmethod could be useful inthe analysis ofchlorantraniliproleresidues in different cereals and vegetable crop ecosystems and application at recommended dose is safe for the final produce at harvest.

## Introduction

Rice is India’s most important food crop for research, production priority and national food security. Repeated use of insecticides throughout the crop growth period has led to contamination in the environment and their residues in crop produce led to consequent health hazard to living organisms^1,2^. The development of environmentally friendly novel molecules to ensure less risk to human health and the environment has led to a better understanding of the possible consequences of such toxic chemicals.

Chlorantraniliprole (CAP) 3-Bromo-N-[4-chloro-2-methyl-6[methylamine]carbonyl] phenyl]-1-(3-chloro-2-pyridinyl)-1Hpyrazole-5carboxamide, a plant systemic insecticide belongs to group anthranilic diamide with a unique mode of action called ryanodine receptor activators that disrupt normal muscle function^3^. Activation of insect ryanodine receptors leads to the unregulated release of calcium (Ca^2+^) from the sarcoplasmic reticulum muscle cells resulting in impaired muscle paralysis, feeding cessation, lethargy, and eventually insect death^4^. CAP was classified as a “reduced-risk pesticide” by the United States Environmental Protection Agency^5^. It is available in two different formulations viz., Chlorantraniliprole 18.5% SC (Suspension Concentrate) and 0.4% G (Granules) recommended at 150 mL ha^−1^ and 10 kg ha^−1^, respectively against yellow rice stem borer and leaf folder management^6^. With exceptional insecticide efficacy, high intrinsic activity on different life stages of insects, no cross-resistance to any current insecticide, low mammalian toxicity, good larvicidal properties, excellent protection profile for honeybees, and other beneficial pollinators, arthropods, soil microorganisms, and earthworms^7^.

CAP studies have so far focused predominantly on chemical synthesis, efficacy, toxicology and mode of action^8–10^. However, many analytical techniques were reported on CAP residue identification and quantification on diverse crop ecosystems viz., fruits, vegetables, pulses and cereals. In grape and tomato, using HPLC with PDA detector^11,12^, cauliflower using HPLC equipped with a mass spectrometer^13^, pigeon pea using LC–MS/MS^14^, corn using UPLC-ESI–MS/MS^15^, maize using UPLC-MS/MS^16^, rice using HPLC–PDA and LC–ESI–MS/MS^17,18^. Determination of various pesticide residues reported in rice^19–22^. The literature reviewed clearly indicated that most of the dissipation studies on CAP were conducted in the vegetable ecosystem^2,12,23–25^. However, a study in the paddy ecosystem using LC–MS/MS in Indian agro-climatic conditions is lacking. Therefore, an attempt is made to develop a highly sensitive and reproducible analytical method involving modified QuEChERS extraction techniques^26,27^ for processing different matrices of paddy such as leaf, straw, husk, brown rice and soil. The present method is highly sensitive with reported LOQ of 0.01 μg g^−1^ (paddy leaf, straw, husk, and brown rice) and 0.005 μg g^−1^ (soil), respectively is the advantages over other techniques in trace level analysis of CAP in the paddy ecosystem. All the matrices in the present study have utility after harvest either as industry products, food grain and animal feed. Further, a supervised field trial was conducted to study the persistence and dissipation of CAP in paddy ecosystem and calculated the half-life, safe waiting period and risk associated with consumption of green leaf by cattle and soil ecological risk on earthworms and arthropods.

## Materials and methods

### Chemicals and reagents

The reference standard of CAP (99.50%) was obtained from Dr.Ehrenstorfer (Augsburg, Germany). A commercial formulation of chlorantraniliprole (CAP) 18.5% SC was procured from the local pesticide shop at Raichur, Karnataka, India. The LC–MS grade organic solvents (methanol and acetonitrile) were purchased from J. T. Baker (NJ, USA) having ≥ 99.50%. HPLC grade water (18.2 MΩ) was collected with a Milli-Q water purification system. Graphitized carbon black (GCB) was procured from Sigma-Aldrich, and analytical grade PSA (Primary secondary amine, 40 μm) was procured from Agilent Technologies India Pvt. Ltd., Bangalore. All the standard chemicals and reagents were procured from Himedia, Bengaluru, India.

### Preparation of standard solutions

A primary standard stock solution of 1000 μg mL^−1^ was prepared using reference material and dissolved in LC–MS grade methanol. Further, working standard solutions of about 10 µg mL^−1^ were prepared from the primary stock solution and checked for linearity study of known concentrations ranging from 0.005 to 1.0 μg mL^−1^. Matrix-matched standards at the same concentrations were prepared by adding approximate CAP solution with control sample extracts of paddy leaf, straw, husk, brown rice and soil separately obtained through the sample preparation procedure described below. All the prepared solutions were placed at − 20 °C until further use.

### Field experiment and sampling

Supervised field trials were conducted at Agricultural Research Station (ARS) Gangavathisituated in the North-eastern dry zone (Zone-3) of Karnataka state (semi-arid eco-sub region) at 15° 15′4 N altitudes and 76° 31′40 E longitudes with an altitude of 419 m above the mean sea level during the *Rabi* (2018–2019) and *Kharif* (2019), with 3 treatments and 8 replications following the Randomized Block Design (RBD). A long-duration paddy variety, “BPT-5204” (145–150 days) was selected for this experiment. All the agronomic practices were performed as and when necessary. Two sprays of CAP 18.5% SC at 30 g a.i. ha^−1^ as Recommended Dose (RD) and 60 g a.i. ha^−1^ as Double the Recommended Dose (DRD) was sprayed between vegetative to tillering stage (60 and 75 days after transplanting) at an interval of 15 days using a high-volume knapsack compression sprayer with a hollow cone nozzle. While spraying, lower dose was sprayed first then the higher dose to avoid cross-contamination of residues. The control plot was sprayed with water. Care was taken to prevent the drift by allowing a buffer zone of 6 feet. Samples of paddy leaf (1 kg) and soil (1 kg) were collected at regular intervals of 0, (2 h after spray) 1, 3, 5, 7, 10, 15, 21, 25, 30, 35 and 40 days after the second application of CAP. Soil samples were collected from the depth of 0–15 cm (10–15 sites) from each replicate plot using a soil auger, pooled and kept in a clean container and extraneous matter (stones/pebbles) were removed. At harvest, paddy grain (1 kg) and straw samples (1 kg) were drawn following the standard sampling procedure^28^. In addition, brown rice samples were obtained by removing the husk from the paddy. Samples collected were carried to the laboratory in dry ice conditions and kept in a freezer at − 20 °C. The collected samples were extracted and analysed using a validated analytical method.

### Extraction and clean up

#### Paddy leaf, straw, brown rice and husk

Method published by Naik et al.^29^ was considered for extraction of different matrices in this study. The paddy leaf samples were mixed, chopped, and macerated thoroughly using a high-volume homogenizer (Robot coup). Straw samples were grounded thoroughly using a high-volume blender and passed through a 2 mm sieve for further analysis. Weighed 5 g sample and transferred into 50 mL polypropylene centrifuge tube and distilled water (10 mL) was added and allowed to stand for 30 min. Then 20 mL acetonitrile was added and vortexed for 1 min. to interact the solvent with the matrix. Next, the sample mixture was homogenized at 10,000–13,000 rpm for 3 min using a low-volume homogenizer.Further, 3 g of sodium chloride was added and vortexed immediately for 2 min and followed by centrifugation at 5000 rpm for 5 min. at 10 °C. After centrifugation, 12 mL of the supernatant was collected in a test tube prefilled with 5 g of sodium sulphate and mixed adequately to remove the moisture content. Then 8 mL of supernatant was transferred to a 15 mL centrifuge tube containing 0.2*g* PSA, 0.4 g anhydrous magnesium sulphate, and 10 mg GCB and vortexed the mixture for 2 min followed by centrifugation at 5000 rpm for 5 min at 10 °C. Later, 1 mL of supernatant was filtered directly using a 0.22 µm PTFE nylon filter to vials for further LC–MS/MS analysis^30^.

#### Soil

Weighed 20 g of sieved soil sample and transferred to 250 mL conical flask followed by addition of 12 mL distilled water and allowed to stand for 30 min. Then, 20 mL of acetonitrile was added and shaken for 4 h at 250 rpm in a shaker. The mixture was then transferred into a 50 mL centrifuge tube and centrifuged at 5000 rpm for 3 min at 10 °C. Later the supernatant was transferred into a 50 mL centrifuge tube containing 6*g* of anhydrous magnesium sulphate, 1.5 g of sodium chloride, 1.5 g of trisodium citrate dihydrate and 750 mg of disodium hydrogen citrate sesquihydrate and vortexed the mixture for 1 min vigorously, followed by centrifugation at 5000 rpm for 5 min at 10 °C. After centrifugation, 6 mL supernatant was transferred into a 15 mL centrifuge tube containing 150 mg PSA and 900 mg anhydrous magnesium sulphate and vortexed for 30 s followed by centrifugation for 5 min at 5000 rpm. Then 3 mL of supernatant was transferred in a test tube and evaporated the content near dryness using a nitrogen flash evaporator at 35 °C. Later reconstituted the residue with 1.5 mL methanol, filtered the content using 0.22 µ PTFE nylon filter into autosampler glass vials for LC–MS/MS analysis.

### Instrumentation

Shimadzu Nexara X2 UHPLC equipment was assembled with solvent reservoir, degassing unit (DGU), LC 30 AD pumps, SIL 30 AC autosampler, and CTO 20 AC column oven was employed for qualitative analysis of the compound using the shimpack XR ODS column at 40 °C. The method was standardized with a mobile phase of ammonium formate (5 mM) solution + 2 mL MeOH + 10 μl of 0.01% formic acid as solvent (A) and ammonium formate (5 mM) + 10 μl formic acid (0.01%) in methanol solvent (B). A gradient mode of mobile phase at 0.3 mL/min flow rate and 2µL of injection volume. With an initial concentration of 95% A and 5% B at the beginning up to 1.20 min. followed by 5% A and 95% B up to 5.00 min. The total run time was 5.0 min.

The mass spectrometry detection was carried out in positive ion mode in LCMS 8040 triple quadrupole equipped with electrospray ionization (ESI +). In multiple reaction monitoring (MRM) mode, estimation was performed to select the most intense mass transition (m/z value). Acquisition parameters for CAP viz., drying gas (15.00 L min^−1^), heat block temperature (400 °C), nebulizing gas flow (2.90 L min^−1^), ion source voltage (4.5 kV), desolvation line temperature (250 °C), injection block temperature (250 °C), and pressure limit of pump A, Pump B (Max 1300 bar, Min 0 bar). For collision, argon gas was used with pressure 230 kpa. Using the program (LabSolution^®^ Version 1.5), instrument control, data recording, and analysis was carried out.

### Method validation

The quantitative analytical method was developed and validated as per SANTE/12,682/2019^31,32^ by ascertaining the following validation parameters viz*.,* specificity, linearity, matrix effect, LOQ, trueness (recovery), precision (repeatability-intraday), precision (reproducibility-interday), ion ratio.

- Specificity: Specificity in the matrix for pesticide residue was evaluated by analysing six individual non-fortified blank samples and compared with the solvent standard for the presence of desired compounds to detect possible interference (Fig. 2).
- Linearity: Linearity check was assessed with a 7-point scale (0.005–1.0 μg mL^−1^) of CAP prepared in solvent standard and control matrix extract and evaluated the deviation of linear concentration injected from the actual linear concentration (residuals) based on the linear regression equation. A linearity graph was drawn by plotting concentrations against the peak area obtained from LC–MS/MS chromatogram, and coefficient of determination (R^2^) was also recorded. Residuals were calculated using the following formula:
  $$ {\text{Deviation}}\,{\text{of}}\,{\text{back}} - {\text{calculated}}\,{\text{concentration}}\,\left( \% \right) = \left( {{\text{C}}_{{{\text{measured}}}} {-}{\text{C}}_{{{\text{true}}}} } \right) \times 100/{\text{C}}_{{{\text{true}}}} $$
  $$ {\text{Measured}}\,{\text{conc}}.\left( {{\text{ng}}\,{\text{g}}^{ - 1} } \right) = \frac{{{\text{Area}}\,{\text{of}}\,{\text{sample}}{-}{\text{intercept}}\left( {\text{c}} \right){\text{of}}\,{\text{linearity}}}}{{{\text{Slope}}\left( {\text{m}} \right){\text{of}}\,{\text{linearity}}}} $$
  $$ {\text{C}}_{{{\text{true}}}} {-}{\text{Linearity}}\,{\text{standard}}\,{\text{concentration}}\left( {{\text{ng}}\,{\text{g}}^{ - 1} } \right) $$
- Matrix effect (ME): Suppression and enhancement of signal is called matrix effect and was evaluated in different extracts to avoid false-positive and false-negative results. The acceptance criteria were ± 20%. The matrix effect was evaluated by using the following formula:
  $$ {\text{Matrix}}\,{\text{effect}}\left( \% \right) = \frac{{{\text{Peak}}\,{\text{area}}\,{\text{of}}\,{\text{matrix}}\,{\text{standard}}{-}{\text{Peak}}\,{\text{area}}\,{\text{of}}\,{\text{solvent}}\,{\text{standard}}}}{{{\text{Peak}}\,{\text{area}}\,{\text{of}}\,{\text{solvent}}\,{\text{standard}}}} \times 100 $$
- LOQ: It was determined by spiking, at the lowest spike level, meeting the method performance criteria for trueness with the recovery of 70–120% and relative standard deviation, RSD ≤ 20%.
- Trueness (Recovery): As per SANTE/12,682/2019 guidelines, 1–10 times of LOQ levels are accepted. Untreated control samples were fortified with CAP at three levels, 0.01, 0.05 and 0.1 μg g^−1^ for paddy leaf, straw, husk and brown rice samples and 0.005, 0.01, 0.02 for soil samples
- Precision (repeatability-intraday): Repeatability test was determined by spiking the CAP standard to the control matrix at 1, 5, and 10 times of LOQ level with six replications and injected three times into LC–MS/MS on the same day. Calculated the obtained concentration from the spiked sample and then calculated percent recovery. The acceptance limit for recovery was 70–120% and RSD ≤ 20%.
- Precision (reproducibility-interday): The method precision was ascertained with regards to the reproducibility of relative standard deviation of the six replicates was carried out by a similar spiking experiment at 1, 5, and 10 times of LOQ level on a subsequent day. Calculated percent recovery and RSD by comparing with a different day of the experiment.
- Ion ratio: Ion ratio was calculated based on the intensity of the quantifier and qualifier ion.

**Figure 1:**
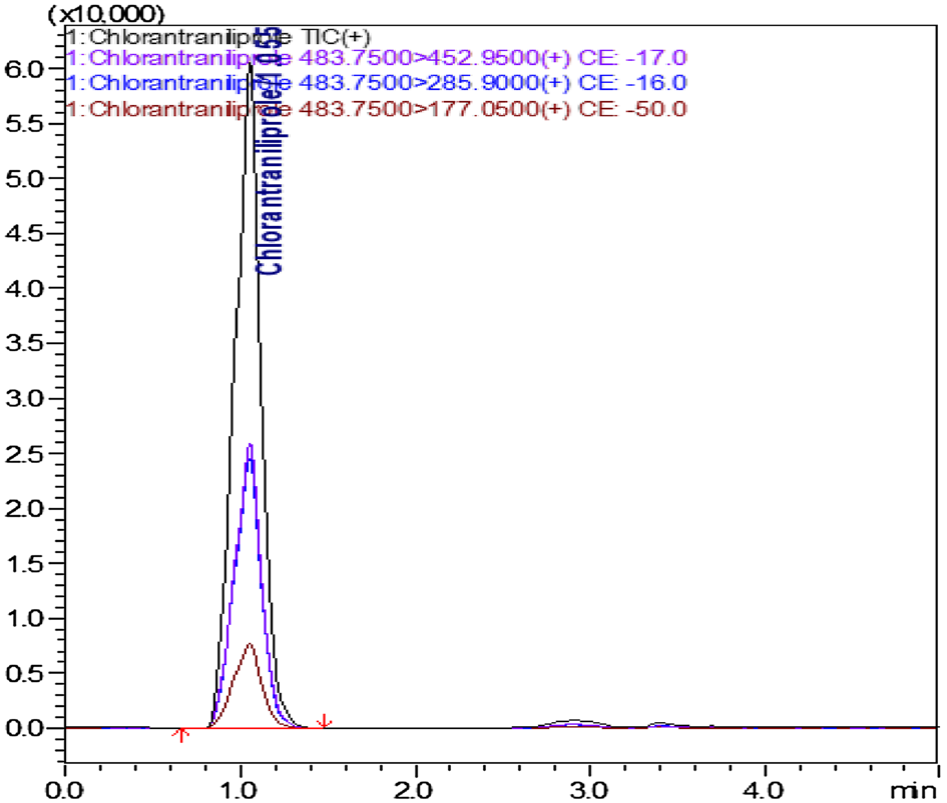
LC–MS/MS determination of chlorantraniliprole along with the MRM at 0.1 µg g^−1^.

**Figure 2:**
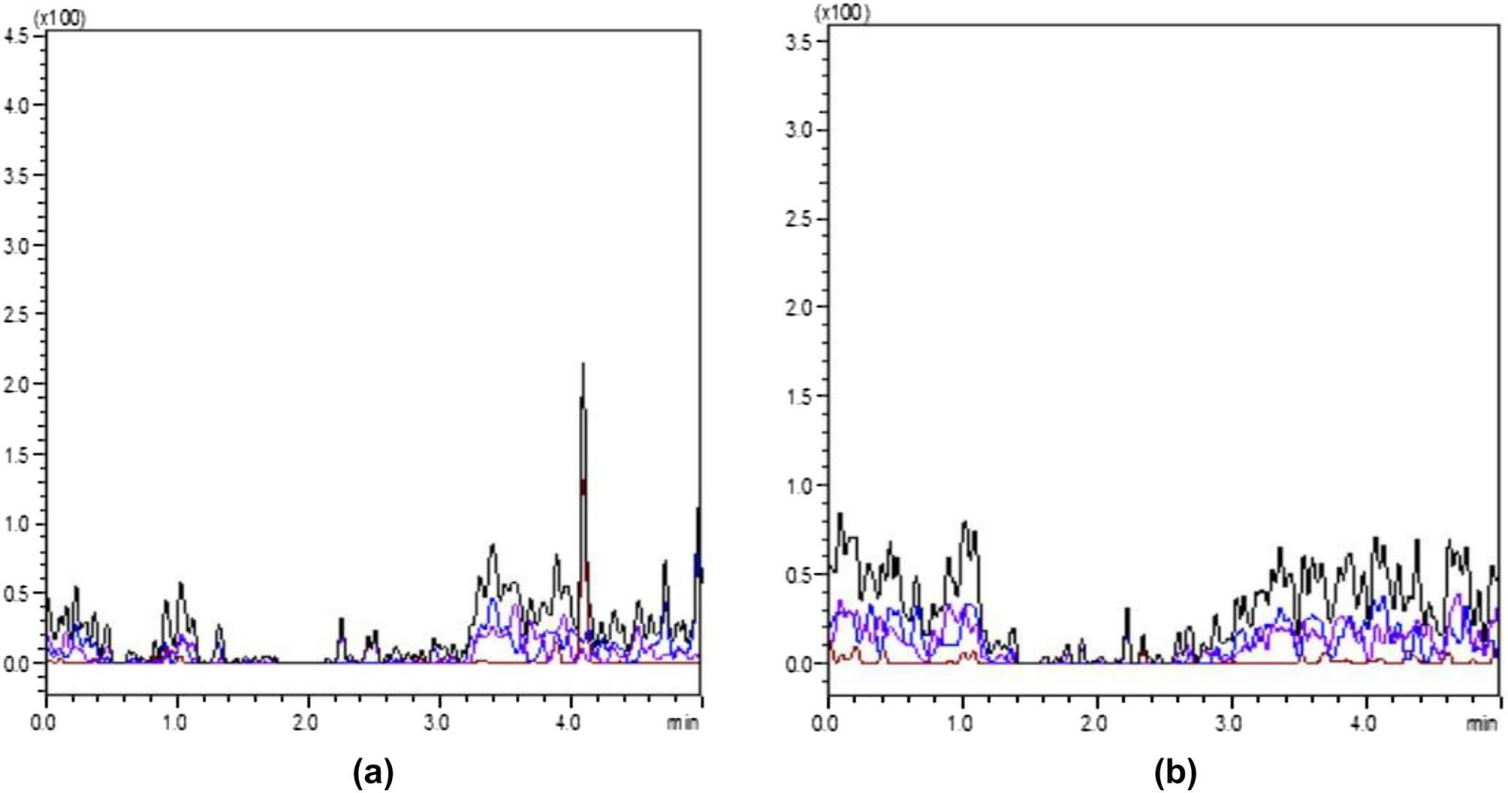
(**a**) LC–MS/MS chromatogram of paddy leaf blank matrix (**b**) paddy soil blank matrix.

### Dietary risk assessment for cattle

Estimated Daily Intake (EDI) was calculated by multiplying the residue concentration (mg/kg) of CAP in paddy leaf obtained under field conditions with the average per capita consumption rate of green fodder (kg animal^−1^ day^−1^), divided by the average bodyweight of the animal. The average per capita consumption rate of green fodder in dry and In-milk cattle was 3.40 and 4.75 kg animal^−1^ day^−1^ as per the NATP project database and the average body weight of dry and In-milk cattle is 245 and 280 kg, respectively^33^. According to the procedure given by the World Health Organization (WHO, Geneva), Hazard Index (HI) was estimated by dividing EDI (mg kg^−1^ day^−1^) by their relevant values of Acceptable Daily Intake (ADI) expressed as mg kg^−1^ body weight (bw) day^−1^. ADI value of CAP is 1.6 mg kg^−1^ day^−1^. If the hazard index value is more than 1, then the food is unfit or not safe for cattle consumption (unacceptable risk) whereas, less than 1 value indicates it is safe for cattle consumption (acceptable risk)^34^.

### Soil ecological risk assessment

In the technical guidance document on risk assessment, the Risk Quotient (RQ) for arthropods (*Aphidius rhopalosiphi*) and earthworms (*Eisenia foetida*) was assessed as specified in the guidelines^35^. The acute 14-day LR_50_ (750 g/ha) value for arthropods (*Aphidius rhopalosiphi*) and LC_50_ (1098 mg/kg) value for earthworms (*Eisenia foetida*) were taken from the Pesticide Properties Database to assess the risk quotient value for arthropods and earthworms^36,37^. By dividing the toxicity with an assessment factor of 1000, the Predicted No-Effect Concentration (PNEC) was calculated. The soil risk quotient was calculated using the formula RQ = EC/PNEC, where EC = Effective Concentration^38^. It indicates low risk if RQ ˂ 0.1, and if RQ values are between 0.1 and 1.0, it indicates moderate risk. RQ of ˃ 1 shows an unacceptable risk of CAP residues in the paddy crop soil^39^.

## Results

### Method validation

**Figure 3:**
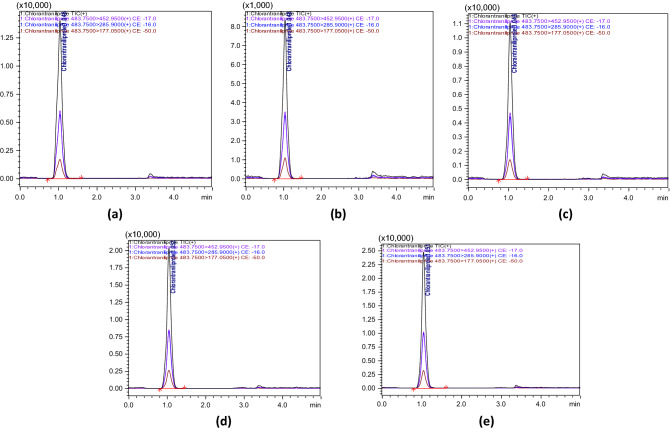
Recovery chromatogram of (**a**) husk at 0.01 µg g^−1^ (**b**) paddy leaf at 0.01 µg g^−1^ (**c**) paddy straw at 0.01 µg g^−1^ (**d**) brown rice at 0.01 µg g^−1^, (**e**) soil at 0.005 µg g^−1^ level of fortification.

The quantitative analytical method was developed and validated as per SANTE/12,682/2019 guidelines^31^.The observed mass transitions and collision energies used in the quantitation of CAP was listed in Table 1. Fragmentation ions at m/z 452.95, 285.90 and 177.05 were observed by the product ion scan of CAP. The most intense transition (m/z) of 452.95 was used for quantification, while the product ion (m/z) of 285.90 and 177.05 were employed for confirmation of CAP residues in samples (Fig. 1). Under the developed method, CAP was found to elute at a retention time of 1.05 ± 0.1 min. The ion ratio between the intensity of the quantification ion and qualifier ions was used as a confirmatory parameter of CAP analysed in selected matrices and ion ratio found to be within the acceptable limit of ± 30% at all spiked concentrations. The linearity of the method showed correlation coefficient (r^2^) of 0.999 for all matrices and 0.998 for solvent standard (Table 2). The matrix effect was 14.42, 18.66, 6.11, 4.39 and 5.78% for leaf, straw, husk, brown rice and soil, respectively (Table 3). The limit of quantification (LOQ) was 0.01 µg g^−1^for paddy leaf, straw, husk, and brown rice, respectively and 0.005 µg g^−1^ for soil (Table 3). The average recoveries obtained were 84.30–88.92% (leaf), 94.25–97.81% (straw), 90.21–93.38% (husk), 93.57–96.40% (brown rice) and 89.93–91.14% (soil) andfulfilled the acceptance criteria of 70–120% recovery and RSD of ≤ 20% (Fig. 3). Percent recoveryin terms of repeatability (same day) and reproducibility (subsequent day) were found within 70–120% and RSD of ≤ 20%. The results are shown in Table 3.

**Table 1:** LC–MS/MS parameters for chlorantraniliprole.

Name of the pesticide	Retention time (min)	Nominal mass (g mol^−1^)	Precursor ion (m/z)	Product ions (m/z)	Collision energy (CE)
Chlorantraniliprole	1.05 ± 0.1	483.10	483.75	4 452.95 (quantifier) 285.90 (confirmation) 177.05 (confirmation)	− 17.0 − 16.0 − 50.0

**Table 2:** The linear regression, coefficient of determination (R^2^) and matrix effect for chlorantraniliprole in paddy leaf and soil.

Parameters	Solvent standard	Chlorantraniliprole				
		Matrix match standards				
		Paddy leaf	Straw	Husk	Brown rice	Soil
Linear regression equation	Y = 24609x − 26,859	Y = 28158x + 34,468	Y = 29201x + 30,388	Y = 26113x + 22,908	Y = 25691x + 12,681	Y = 26032x + 28,874
Coefficient of determination (R^2^)	0.998	0.999	0.999	0.999	0.999	0.999
Limit of detection (µg g^−1^)	0.00025	0.00025	0.00025	0.00025	0.00025	0.00025
Limit of quantification (µg g^−1^)	–	0.01	0.01	0.01	0.01	0.005
Matrix effect (%)		14.42	18.66	6.11	4.39	5.78

**Table 3:** The recovery of chlorantraniliprole in paddy leaf, straw, husk, brown rice and soil.

Spiked level (µg g^-1^)	Recovery ± RSD % (n = 6)				Spiked level (µg g^-1^)	Recovery ± RSD % (n = 6)
	Paddy leaf	Straw	Husk	Brown rice		Paddy soil
Trueness						
0.01	88.92 ± 7.24	97.81 ± 3.80	90.21 ± 3.32	96.40 ± 5.49	0.005	89.93 ± 5.46
0.05	86.15 ± 5.38	94.25 ± 3.00	93.38 ± 3.98	93.57 ± 3.63	0.01	90.94 ± 5.18
0.1	84.30 ± 6.13	95.08 ± 4.52	92.26 ± 5.84	95.04 ± 4.48	0.02	91.14 ± 5.37
Intraday precision (repeatability-RSDr)						
0.01	93.85 ± 4.55	90.68 ± 5.39	91.47 ± 5.37	97.71 ± 2.69	0.005	95.84 ± 2.18
0.05	97.08 ± 4.97	91.31 ± 3.99	90.70 ± 5.89	95.51 ± 4.51	0.01	96.58 ± 2.63
0.01	101.92 ± 8.45	88.20 ± 3.87	94.99 ± 3.59	99.15 ± 2.33	0.02	98.47 ± 4.44
Intraday precision (reproducibility-RSDwr)						
0.01	94.89 ± 5.30	97.29 ± 3.64	94.76 ± 6.05	93.14 ± 4.15	0.005	93.27 ± 3.36
0.05	99.83 ± 4.87	98.11 ± 4.51	96.78 ± 4.66	94.47 ± 5.19	0.01	92.99 ± 3.95
0.01	100.15 ± 6.62	96.45 ± 4.58	97.64 ± 4.08	96.19 ± 4.47	0.02	90.22 ± 4.26

### Dissipation study

CAP residues in paddy leaf and soil obtained from field trials were subjected to first-order dissipation kinetics equation, i.e., Ct = Coe^−kt^, Ct is the concentration of pesticide (μg g^−1^) at time t (day), Co is the apparent initial concentration after application (μg g^−1^) and k is the degradation rate constant^30^. CAP’s half-life (T1/2) was calculated as T_1/2_ = log2/k^40^. T_1/2_ is the insecticide half-life in paddy leaves and soil.

### CAP residues in paddy leaf

Initial deposits, degradation dynamics and half-life of CAP in paddy leaf for both *Rabi* and *Kharif* were given in Table 2. There is a rapid loss of CAP residues within the first 24 h after treatment and subsequently, a slower dissipation rate was observed (Table 4). The mean initial deposition of CAP was higher in *Rabi* (2.599 µg g^−1^) compared to *Kharif* (2.347 µg g^−1^) at 30 g a.i.ha^−1^. Similarly, at 60 g a.i.ha^−1^ also residue deposition is higher in *Rabi* (5.975 µg g^−1^) than *Kharif* (5.680 µg g^−1^).During rabi, residues were degraded from 1.988 to 0.023 and 4.735 to 0.047 µg g^−1^, accounting for 99.12 and 99.23% loss, respectively in RD and DRD. Similarly, during *Kharif,* residues were gradually dissipated from 1.852 to 0.012 and 4.620 to 0.017 µg g^−1^after application, accounting for the loss of 99.49 and 99.70%, respectively in RD and DRD. The residues in both seasons reached below the quantification level (0.01 µg g^−1^) on 35th and 40th day after application at RD and DRD, respectively. Various factors viz*.,* pesticide and its formulation, concentration of an active ingredient, substrate characteristics, plant type, growth of plant part, shape of the plant and weather parameters such as relative humidity, temperature, precipitation and wind movement influence the initial deposit of residues in both seasons^30^. The half-life recorded in paddy leaf was 4.33 days (Ct = 2.2179e^−0.160t^) (RD) and 5.07 days (Ct = 6.1144e^−0.137t^) (DRD) in *Rabi* and 3.92 (Ct = 2.419e^−0.177t^) days (RD) and 4.86 (Ct = 5.8263e^−0.143t^) days (DRD) in *Kharif* at 30 and 60 g a.i. ha^−1^, respectively. The residues at the time of harvest in brown rice, husk and straw were below the detectable limit (< 0.01 µg g^−1^) in both the doses applied, indicates application of CAP is safe from residues point of view.

### CAP residues in soil

**Figure 4:**
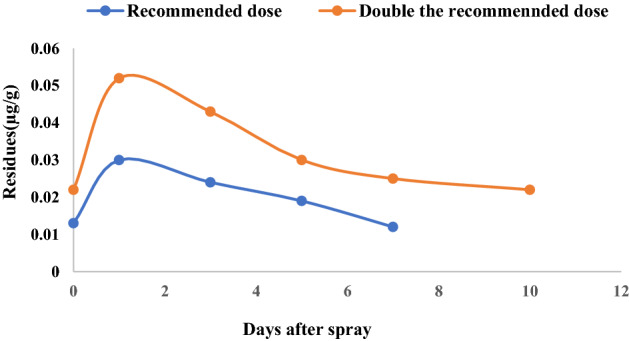
Dissipation of chlorantraniliprole 18.5% SC in paddy soil during rabi at recommended dose and double the recommended dose.

**Figure 5:**
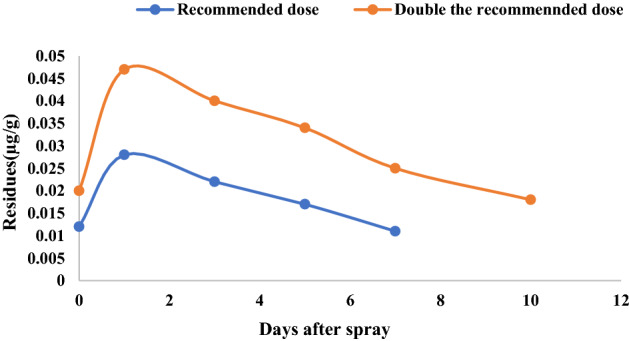
Dissipation of chlorantraniliprole 18.5% SC in paddy soil during kharif at recommended dose and double the recommended dose.

Average residues of CAP in soil were 0.013, 0.030, 0.024, 0.019 and 0.012 µg g^−1^ after 0 (2 h), 1, 3, 5 and 7 days, respectively for RD and 0.022, 0.052, 0.043, 0.030, 0.025 and 0.022 for DRD after 0 (2 h), 1, 3, 5, 7 and 10 days, respectively after second spray during *Rabi* season (Fig. 4). During *Kharif* season average residues of CAP in soil were 0.012,0.028, 0.022, 0.017 and 0.011 µg g^−1^ after 0 (2 h), 1, 3, 5 and 7 days, respectively for RD and 0.020, 0.047, 0.040, 0.034, 0.025 and 0.018 for DRD after 0 (2 h), 1, 3, 5, 7 and 10 days, respectively (Fig. 5). The present results showed a sharp rise in CAP residues from 2 h to 1 day in both *Rabi* and *Kharif.*Residues of CAP during *Rabi* dissipated to half of its concentration (T_1/2_) on 14.44 days (Ct = 0.0215e^0.048t^) and 15.75 days (Ct = 0.0371e^0.044t^) and during *Kharif* the half-life was 13.33 days (Ct = 0.0202e^0.052t^) and 14.44 days (Ct = 0.036e^0.048t^) for RD and DRD, respectively.

### Dietary risk assessment in cattle

The residue dissipation data are used for calculating the risk assessment of CAP applied in paddy leaf under open field conditions. The calculated hazard index (HI) reflected less than one from 0 days (after 2 h) after the application of CAP, irrespective of doses in both seasons (Tables 5 and 6) indicating paddy leaf was safe for consumption in both dry and In milk cattle.

**Table 4:** Persistence of chlorantraniliprole 18.5%SC in paddy leaf at recommended and double the recommended dose.

Days after treatment	*Rabi* season2018–2019				*Kharif* season2019–2020			
	Recommended dose (30 g a.i ha^−1^)		Double the recommended dose (60 g a.i ha^−1^)		Recommended dose (30 g a.i ha^−1^)		Double the recommended dose (60 g a.i ha^−1^)	
	Residue (µg g^−1^)^a^ (Mean ± SD)	Dissipation (%)	Residue (µg g^−1^)^a^ (Mean ± SD)	Dissipation (%)	Residue (µg g^−1^)^a^ (Mean ± SD)	Dissipation (%)	Residue (µg g^−1^)^a^ (Mean ± SD)	Dissipation (%)
0 (2 h)	2.599 ± 0.182	–	5.975 ± 0.511	–	2.347 ± 0.202	–	5.680 ± 0.053	–
1	1.988 ± 0.086	23.51	4.735 ± 1.068	20.75	1.852 ± 0.115	21.09	4.620 ± 0.005	18.66
3	1.526 ± 0.116	41.29	3.644 ± 1.110	39.01	1.521 ± 0.054	35.19	3.874 ± 0.589	31.80
5	1.057 ± 0.266	59.33	3.324 ± 0.549	44.35	0.854 ± 0.048	63.61	2.583 ± 0.387	54.52
7	0.744 ± 0.106	71.37	2.345 ± 0.697	60.74	0.687 ± 0.051	70.73	1.706 ± 0.012	69.96
10	0.323 ± 0.094	87.53	1.543 ± 0.209	74.19	0.456 ± 0.039	80.57	1.201 ± 0.006	78.86
15	0.160 ± 0.011	93.84	0.870 ± 0.342	85.44	0.252 ± 0.114	89.26	0.757 ± 0.081	86.67
21	0.060 ± 0.014	97.69	0.451 ± 0.082	92.47	0.054 ± 0.008	97.70	0.525 ± 0.060	90.76
25	0.047 ± 0.010	98.19	0.245 ± 0.133	95.90	0.025 ± 0.013	98.93	0.278 ± 0.039	95.11
30	0.023 ± 0.013	99.12	0.074 ± 0.023	98.76	0.012 ± 0.006	99.49	0.099 ± 0.004	98.26
35	BDL	–	0.047 ± 0.021	99.23	BDL	–	0.017 ± 0.003	99.70
40	–	–	BDL	–	–	–	BDL	–
Rice grain (At Harvest)	BDL		BDL		BDL		BDL	
Rice husk (At Harvest )	BDL		BDL		BDL		BDL	
Paddy straw (At Harvest)	BDL		BDL		BDL		BDL	
Correlation coefficient (R^2^) = 0.987 Kinetic equation Ct = 2.2179e^-0.160x^ T_1/2_ = 4.33 days			Correlation coefficient (R^2^) = 0.991 Kinetic equation Ct = 6.1144e^-0.137t^ T_1/2_ = 5.07 days		Correlation Coefficient (R^2^) = 0.993 Kinetic equation Ct = 2.419e^-0.177t^ T_1/2_ = 3.92 days		Correlation Coefficient (R^2^) = 0.954 Kinetic equation Ct = 5.8263e^-0.143t^ T_1/2_ = 4.86 days

**Table 5:** Dietary risk assessment of chlorantraniliprole 18.5% WG in cattles during *Rabi,* 2018–2019.

Daysafter application	*Rabi*, 2018–2019										
	Residue (µg g^−1^) 30 g a.i.ha^−1^	ADI (mg kg^−1^ day^−1^)	EDI (kg animal^−1^ day^-1^)		Hazard index (HI)		Residue (µg g^−1^) 60 g a.i. ha^−1^	EDI (kg animal^−1^ day^−1^)		Hazard index (HI)	
			In milk cattle	Dry cattle	In milk cattle	Dry cattle		In milk cattle	Dry cattle	In milk cattle	Dry cattle
0 (2 h)	2.599	1.6	0.0441	0.0361	0.0276	0.0225	5.978	0.1014	0.0829	0.0634	0.0518
1	1.988	1.6	0.0337	0.0276	0.0211	0.0172	4.735	0.0803	0.0657	0.0502	0.0411
3	1.526	1.6	0.0259	0.0212	0.0162	0.0132	3.644	0.0618	0.0506	0.0386	0.0316
5	1.057	1.6	0.0179	0.0147	0.0112	0.0092	3.324	0.0564	0.0461	0.0352	0.0288
7	0.744	1.6	0.0126	0.0103	0.0079	0.0065	2.345	0.0398	0.0325	0.0249	0.0203
10	0.323	1.6	0.0055	0.0045	0.0034	0.0028	1.543	0.0262	0.0214	0.0164	0.0134
15	0.160	1.6	0.0027	0.0022	0.0017	0.0014	0.870	0.0148	0.0121	0.0092	0.0075
21	0.060	1.6	0.0010	0.0008	0.0006	0.0005	0.451	0.0077	0.0063	0.0048	0.0039
25	0.047	1.6	0.0008	0.0007	0.0005	0.0004	0.245	0.0042	0.0034	0.0026	0.0021
30	0.023	1.6	0.0004	0.0003	0.0002	0.0002	0.074	0.0013	0.0010	0.0008	0.0006
35	–	–	–	–	–	–	0.047	0.0008	0.0007	0.0005	0.0004

*ADI* acceptable daily intake, *EDI* estimated daily intake.

**Table 6:** Dietary risk assessment of chlorantraniliprole 18.5% WG in cattle’s during kharif, 2019–2020.

Days after application	Kharif, 2019–2020										
	Residue (µg g^−1^) 30 g a.i. ha^−1^	ADI (mg kg^−1^ day^−1^)	EDI (kg animal^−1^ day^−1^)		Hazard index (HI)		Residue (µg g^−1^) 60 g a.i. ha^−1^	EDI (Kg animal^−1^ day^−1^)		Hazard index (HI)	
			In milk cattle	Dry cattle	In milk cattle	Dry cattle		In milk cattle	Dry cattle	In milk cattle	Dry cattle
0 (2 h)	2.347	1.6	0.0398	0.0326	0.0249	0.0204	5.680	0.0964	0.0788	0.0602	0.0493
1	1.852	1.6	0.0314	0.0257	0.0196	0.0161	4.620	0.0784	0.0641	0.0490	0.0401
3	1.521	1.6	0.0258	0.0211	0.0161	0.0132	3.874	0.0657	0.0538	0.0411	0.0336
5	0.854	1.6	0.0145	0.0119	0.0091	0.0074	2.583	0.0438	0.0358	0.0274	0.0224
7	0.687	1.6	0.0117	0.0095	0.0073	0.0060	1.706	0.0289	0.0237	0.0181	0.0148
10	0.456	1.6	0.0077	0.0063	0.0048	0.0040	1.201	0.0204	0.0167	0.0127	0.0104
15	0.252	1.6	0.0043	0.0035	0.0027	0.0022	0.757	0.0128	0.0105	0.0080	0.0066
21	0.054	1.6	0.0009	0.0007	0.0006	0.0005	0.525	0.0089	0.0073	0.0056	0.0046
25	0.025	1.6	0.0004	0.0003	0.0003	0.0002	0.278	0.0047	0.0039	0.0029	0.0024
30	0.012	1.6	0.0002	0.0002	0.0001	0.0001	0.099	0.0017	0.0014	0.0010	0.0009
35	–	–	–	–	–	–	0.017	0.0003	0.0002	0.0002	0.0001

*ADI* acceptable daily intake, *EDI* estimated daily intake.

### Soil ecological risk assessment

The RQ of CAP in soil indicated a low (RQ ˂ 0.1) risk to arthropodaand earthworm at both the doses after application (Table 7). Similar results were observed when CAP was applied to tomato field soil and okra field soil^2,25^.

**Table 7:** Ecological risk assessment of chlorantraniliprole 18.5% SC in soil.

DAT	Residues (µg g^−1^)					Soil ecological risk assessment								
	Rabi, 2018–2019		Kharif, 2019–2020		PNEC (mg/kg)	Earthworm (*Eisenia foetida*)				PNEC (mg/kg)	Arthropoda (*Aphidiusrhopalosiphi*)			
	RD	DRD	RD	DRD		Rabi, 2018–2019		Kharif, 2019–2020			Rabi, 2018–2019		Kharif, 2019–2020	
						RD	DRD	RD	DRD		RD	DRD	RD	DRD
						RQ	RQ	RQ	RQ		RQ	RQ	RQ	RQ
0 (2 h)	0.013	0.022	0.012	0.020	1.00	0.01	0.02	0.01	0.02	0.75	0.02	0.03	0.02	0.03
1	0.030	0.052	0.028	0.047	1.00	0.03	0.05	0.03	0.05	0.75	0.04	0.07	0.04	0.06
3	0.024	0.043	0.022	0.040	1.00	0.02	0.04	0.02	0.04	0.75	0.03	0.06	0.03	0.05
5	0.019	0.030	0.017	0.034	1.00	0.02	0.03	0.02	0.03	0.75	0.03	0.04	0.02	0.05
7	0.012	0.025	0.013	0.025	1.00	0.01	0.03	0.01	0.03	0.75	0.02	0.03	0.02	0.03
10	0.007	0.023	0.011	0.021	1.00	0.00	0.02	0.01	0.02	0.75	0.01	0.03	0.01	0.03
15	–	0.019	–	0.016	1.00	–	0.01	–	0.02	0.75	–	0.03	–	0.02

*DAT* days after treatment, *RD* recommended dose (30 g a.i./ha), *DRD* double the recommended dose (60 g a.i./ha), *PNEC* predicted no effect concentration, *RQ* risk quotient.

## Discussion

In the present study, LC mass spectrometry analytical method was developed and validated for CAP in different matrices of paddy (leave, husk, straw, brown rice and soil). Recovery of CAP in different matrices was in the range of 70–120% with satisfactory precision (RSD ≤ 20%). Dissipation study of CAP in paddy leaf during both the season shows a difference in the residue deposition which may be due to various chemical and physical factors that play a major role in pesticide degradation viz*.,* light, heat, moisture, and pH^15,16^. Residues of CAP in paddy leaf dissipated over time, although the loss of applied pesticide may also depend on the dilution caused due to the growth of the treated plant. The half-life of CAP in rice straw (3.50 days) was observed in Zhejiang province of China^18^; 3.2, 4.4 and 6.3 days (rice straw) in Zhejiang, Hunan and Shandong regions of China^41^, in corn straw 4.9 and 5.4 days in Henan and Shandong region of China^15^. Bhardwaj et al.^17^reported residues of CAP in basmati rice plants was found to be below determination limit of 0.05 mg kg^−1^ at 15 and 20 days after application and did not reveal the presence of CAP residues in basmati grains, bran, husk and straw at harvest. Vijayasree et al.^23^ reported initial deposits of CAP in cowpea fruits were found to be 0.55 mg kg^−1^@ 30 g a.i. ha^−1^ and reached below determination limit of 0.05 mg kg^−1^ at 10 days after application of the pesticide. Initial residues of CAP at single (30 g a.i. ha^−1^) and double doses (60 g a.i. ha^−1^) on the fruits of brinjal were 0.72 and 1.48 mg kg^−1^, while on okra fruits, the residues were 0.48 and 0.91 mg kg^−1^, respectively. The residues reached below detectable level of 0.01 mg kg^−1^ on the 10th day^24^. The initial deposits of CAP were 0.18 and 0.29 mg kg^−1^ on cauliflower curds @ 9.25 and 18.5 g a.i. ha^−1^, respectively and reached below the quantification level of 0.05 mg kg^−1^ in 3 and 5 days, respectively^13^. Malhat^11^reported the persistence of CAP residues in grape fruits with average initial deposit of 2.829 mg kg^−1^ @ 60 mL per feddan and dissipated to undetectable limit 21 days after the treatment. CAP residues in tomato fruit with initial deposits of 2.30 mg kg^−1^ following @ 60 mL per feddan, decreased to 1.712 mg kg^−1^ within 24 h, after application was reported and residue, reached below the quantification limit of 0.03 mg kg^−1^ at 21 days after application^12^. In another study, residues of CAP were dissipated to below the detectable level at 10 days after the second spray with a half-life of 2.21 and 1.26 days in okra and tomato fruits, respectively^2,25^.The present discussion strongly indicates less persistence nature of CAP in the vegetable ecosystem compared to the paddy ecosystem.

In the dissipation study of soil, a sharp rise in CAP residues from 2 h to 1 day may be due to the chemical properties of the CAP contributing to the adsorption equilibrium period in the paddy field ecosystem between water and soil over 2 h. The strong adsorption of CAP in the soil may be due to the higher organic matter content of the soil and pH. The organic matter and pH of the studied soil was 1.53% and 7.82, respectively in the present study. It can be observed that the major degradation of CAP took place within the first week after application. However, during the succeeding days, deterioration occurred at a slower rate. Zhang et al.^18^ also observed a sharp increase in the CAP residues that occurred from 2 to 8 h in the paddy soil. Zhang et al.^18^ found the half-life of CAP in paddy soil was 16.0 dayswhereas, Malhat et al.^12^ reported half-life (t_1/2_) value of 3.66 days for CAP in tomato soil. The dissipation rate in paddy soil was significantly lower than that of the vegetable ecosystem (half-life of 9.0–10.7 days)^42^.The terminal residues in paddy soil at the time of harvest in both seasons were below the detectable limit (< 0.005 µg g^−1^) irrespective of the doses applied.

## Conclusion

In conclusion, an efficient, sensitive and simple LC-MS/MS method was developed and validated successfully (recovery-70–120% and RSD ≤ 20 %) to determine CAP residues in the paddy ecosystem. The study's findings revealed that CAP at 30 and 60 g a.i. ha^−1^ is short-lived in the paddy ecosystem and did not record residues in the straw and harvested grains. So, it confirms that two applications at recommended rate could be appropriate to manage key insect pests and did not leave any residues in the final product. This proposed method could be useful in monitoring CAP residues in other cereals and vegetable crops meant for consumption.

## Data Availability

The datasets generated during and/or analysed during the current study are available from the corresponding author on reasonable request.
